# Dyspnea-Related Cues Engage the Prefrontal Cortex: Evidence From Functional Brain Imaging in COPD

**DOI:** 10.1378/chest.15-0416

**Published:** 2015-07-02

**Authors:** Mari Herigstad, Anja Hayen, Eleanor Evans, Frances M. Hardinge, Robert J. Davies, Katja Wiech, Kyle T. S. Pattinson

**Affiliations:** From the Oxford Centre for Functional MRI of the Brain (FMRIB) (Drs Herigstad, Hayen, Wiech, and Pattinson and Ms Evans), Nuffield Department of Clinical Neurosciences, University of Oxford, Oxford; Department of Clinical Health Care (Dr Herigstad), Oxford Brookes University, Oxford; School of Psychology and Clinical Language Sciences (Dr Hayen), University of Reading, Reading; and Oxford Respiratory Trials Unit (Drs Hardinge and Davies), Nuffield Department of Medicine, University of Oxford, Oxford, England.

## Abstract

**BACKGROUND:**

Dyspnea is the major source of disability in COPD. In COPD, environmental cues (eg, the prospect of having to climb stairs) become associated with dyspnea and may trigger dyspnea even before physical activity commences. We hypothesized that brain activation relating to such cues would be different between patients with COPD and healthy control subjects, reflecting greater engagement of emotional mechanisms in patients.

**METHODS:**

Using functional MRI (FMRI), we investigated brain responses to dyspnea-related word cues in 41 patients with COPD and 40 healthy age-matched control subjects. We combined these findings with scores on self-report questionnaires, thus linking the FMRI task with clinically relevant measures. This approach was adapted from studies in pain that enabled identification of brain networks responsible for pain processing despite absence of a physical challenge.

**RESULTS:**

Patients with COPD demonstrated activation in the medial prefrontal cortex and anterior cingulate cortex, which correlated with the visual analog scale (VAS) response to word cues. This activity independently correlated with patient responses on questionnaires of depression, fatigue, and dyspnea vigilance. Activation in the anterior insula, lateral prefrontal cortex, and precuneus correlated with the VAS dyspnea scale but not with the questionnaires.

**CONCLUSIONS:**

The findings suggest that engagement of the emotional circuitry of the brain is important for interpretation of dyspnea-related cues in COPD and is influenced by depression, fatigue, and vigilance. A heightened response to salient cues is associated with increased symptom perception in chronic pain and asthma, and the findings suggest that such mechanisms may be relevant in COPD.

Dyspnea causes immense suffering for patients with COPD. Objective measures of lung function, such as spirometry, correlate poorly with dyspnea.^1^ Despite dyspnea being subjective, it remains the best predictor of mortality.^2^ There is a clear need to better understand the mechanisms of dyspnea to find new treatments.

Contemporary models emphasize that the experience of dyspnea is strongly influenced by psychologic processes, particularly depression^3‐5^ and dyspnea-related fear and anxiety.^5‐9^ Although the physical sensation of dyspnea commonly originates from sensory afferent sources, including the heart, lungs, and muscles, conscious awareness of dyspnea arises in the brain.^5‐8^ Repeated association between environmental cues and dyspnea may increase the salience of such cues. For example, a ringing telephone could be associated with the need to move quickly to answer it and, thus, may trigger brain anticipatory dyspnea circuitry even before physical activity commences. One way of measuring the activity in these brain circuits is with functional MRI (FMRI).^10^

In this study, FMRI was used to investigate brain processes associated with responses to dyspnea-related cues in COPD. We adapted methodologies in which salient images or word cues engage pain processing networks in the brain despite the absence of a physical challenge.^11,12^ Brain state before a stimulus is known to influence subsequent perception.^13^ We hypothesized that differences in brain activation to dyspnea-related environmental cues between patients with COPD and healthy control subjects may reflect differences in salience of these cues and changes in the cognitive-affective state.

## Materials and Methods

### Participants

We recruited 41 patients (15 women; mean ± SD age, 68.0 ± 8.2 years) with mild to moderate COPD (according to GOLD [Global Initiative for Chronic Obstructive Lung Disease] criteria) in the week before commencing a course of pulmonary rehabilitation and 40 age- and sex-matched healthy control subjects (16 women; mean age, 69.1 ± 8.1 years). Demographics are shown in Table 1; medical details and recruitment procedures are provided in e-Appendix 1, e-Figure 1, and e-Table 1. All participants gave written informed consent. The study was approved by Oxfordshire Research Ethics Committee A (09/H0604/108).

**TABLE 1 ]:** Participant Details and Physiologic Data

Demographic	Patients	Control Subjects
Age, y	68.0 ± 8.2	69.1 ± 8.1
Male (female) sex	15 (26)	16 (24)
IMD score	12.1 ± 6.8	11.7 ± 9.2
BMI, kg/m^2^	28.4 ± 6.7	25.2 ± 3.2^a^
MRC breathlessness score (1-5)	3 (IQR 2-4)	1 (IQR 1-1)^b,c^
GOLD stage (0-IV)	2 (IQR 1-3)	…
Resting Borg score (1-10)	0.8 ± 1.1	0.06 ± 0.2^b^
Resting Sao_2_, %	94.4 ± 2.6	96.4 ± 1.3^b^
Resting heart rate, beats/min	82.8 ± 13.7	72.2 ± 11.0^b^
MSWT distance, m	320 ± 185	804 ± 274^b^
Smoking history, pack-y	40.3 ± 33.3	3.1 ± 6.6^b^
FEV_1_, % predicted	58 ± 21	99 ± 24^b^

Data are presented as mean ± SD, No., or median (IQR). GOLD = Global Initiative for Chronic Obstructive Lung Disease; IMD = English Indices of Deprivation, 2010 data; IQR = interquartile range; MRC = Medical Research Council; MSWT = modified shuttle walk test; Sao_2_ = oxygen saturation as measured by pulse oximetry.

a *P* < .01.

b *P* < .001.

c Mann-Whitney-Wilcoxon test.

### Functional MRI

**Figure 1 –:**
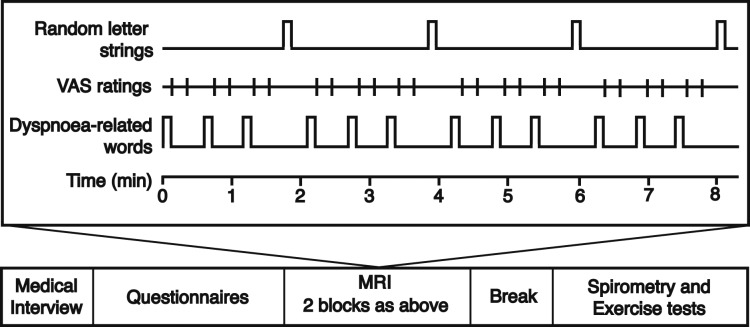
Functional MRI task and protocol. Participants were presented with and asked to rate dyspnea-related word cues. Dyspnea-related word cues and ratings were each displayed for 7 s. Every block was separated by a fixation cross (12 s), and every third block was followed by random letter strings (7 s). VAS = visual analog scale.

Imaging was performed with a 3T MAGNETOM Trio, A Tim System (Siemens Healthcare GmbH) using a 12-channel head coil. Participants underwent two FMRI scans (each 8 min, 20 s, with a 30-s break between) and one structural scan (Fig 1).

**Figure 2 –:**
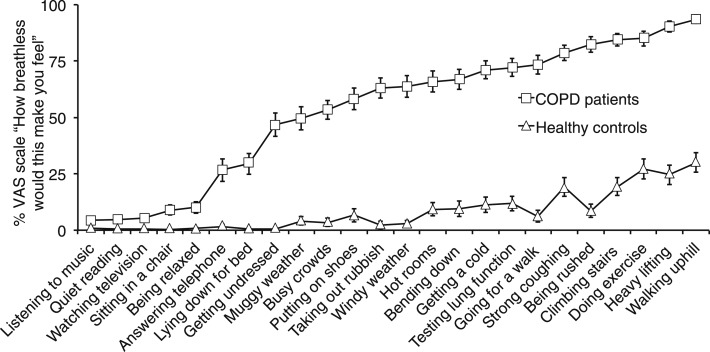
Mean VAS responses to the question, “How breathless would this make you feel,” in 41 patients with COPD and 40 healthy age- and sex-matched control subjects. Anchors were not at all and very much. Error bars represent SEM. See Figure 1 legend for expansion of abbreviation.

During scanning, participants were shown a randomized set of dyspnea-related word cues. These cues were designed to induce recall of everyday situations that may be associated with dyspnea in patients with COPD, ranging from low to high valence (Fig 2). Participants viewed each word and rated it using a button box according to how breathless and how anxious it would make them feel on a visual analog scale (VAS) of 0 to 10 (anchors not at all and very much) presented on-screen. To familiarize themselves with the protocol before scanning, participants completed a set of eight test words.

Heart rate, oxygen saturation as measured by pulse oximetry (Sao_2_) as measured by a MultiGas Monitor 9500 (MR Equipment), respiration (chest respiratory bellows), and end-tidal Po_2_ and Pco_2_ (Normocap 200 Capnometer [Datex Division Instrumentation Corp]; nasal cannula [Salter Labs]) were continuously measured throughout the scan. All physiologic data were sampled at 50 Hz and recorded along with scan volume triggers through PowerLab 8 using Chart 5 (ADInstruments).

### Psychologic Measurements

Before scanning, a comprehensive assessment of respiratory perception and impact was obtained using the following self-report questionnaires: St. George’s Respiratory Questionnaire,^14^ Medical Research Council dyspnea scale,^15^ Dyspnea-12 questionnaire,^16^ and Catastrophizing about Asthma Scale^17^ and Pain Vigilance and Awareness Questionnaire^18^ (modified by substituting the word “breathlessness” for the words “asthma” and “pain,” respectively). Depression, anxiety, fatigue, and demotivation are important coexisting psychologic processes in COPD and were measured with the Center for Epidemiologic Studies Depression Scale,^19^ State-Trait Anxiety Inventory,^20^ Fatigue Severity Scale,^21^ and Behavioral Inhibition System/Behavioral Activation System scale.^22^

### Spirometry and Exercise Testing

Participants underwent spirometry performed by a trained respiratory nurse using Association for Respiratory Technology and Physiology standards^23^ and a modified shuttle walk test (MSWT) performed twice. Before, during, and after the MSWT, heart rate and Sao_2_ were measured every minute using a fingertip pulse oximeter (Go_2_; Nonin Medical Inc). Participants rated their dyspnea on a modified Borg scale immediately before and after the MSWT (e-Table 2).

### Data Analysis

All FMRI data processing was carried out with FEAT (FMRI Expert Analysis Tool) version 5.98 (FMRIB Software Library [www.fmrib.ox.ac.uk/fsl]) using a whole-brain approach with standard parameters. First-level analyses used a general linear model with multiple explanatory variables, which were presentation of word cues, trial-by-trial dyspnea and anxiety ratings of word cues, random letter strings, and periods when subjects were rating using the VAS. Physiologic noise correction was performed using RETROICOR (retrospective image correction).^24,25^

A multiple regression analysis using SPSS Statistics software (IBM Corporation) was performed for all questionnaire scores to identify the major psychologic factors contributing to the Dyspnea-12 scores (dependent variable). The following scores were identified and included as additional regressors in the higher-level analysis: state anxiety, fatigue, depression, and awareness and vigilance.

A higher (group)-level analysis was performed. Because fatigue, depression, and vigilance are known to be major factors in dyspnea, these were considered regressors of interest. Because state anxiety may have been confounded by experimental factors, this was considered a regressor of no interest. Because we had no prior expectation of any link between these factors and breathlessness in control subjects, these regressors were not interrogated further in the control group. To correct for multiple comparisons, *z*-statistic image thresholds were set using clusters determined by *z* > 2.3 and a (corrected) cluster significance threshold of *P* = .05 across the whole brain.^26^

We performed conjunction analyses (conjunction null) to determine common areas of mean brain activation in each group and among the questionnaire regressors in patients only. Unpaired *t* tests determined between-group differences. *F* tests were performed to test for shared variance between the different variables.

Questionnaires were scored according to their respective manuals. For spirometry, the measurement associated with the highest FEV_1_ value was used. For MSWT, the measurement associated with the farthest distance walked was used. Data were compared using Student *t* test. Correlations among FEV_1_, MSWT, and Dyspnea-12 scores were assessed with MATLAB software (MathWorks, Inc), and *P* < .017 (Bonferroni corrected) were considered significant. A detailed description of study methods can be found in e-Appendix 1.

## Results

### Participants

**Figure 3 –:**
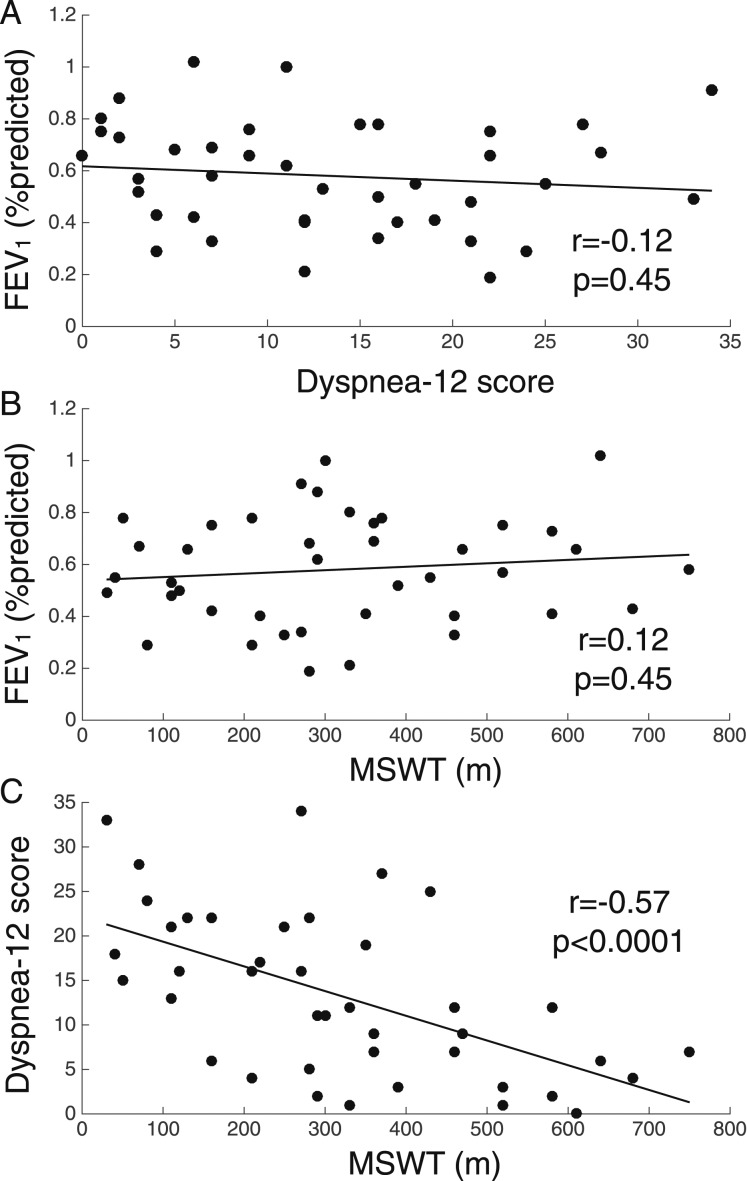
A-C, FEV_1_, exercise performance, and dyspnea in patients with COPD. Percent predicted FEV_1_ plotted against Dyspnea-12 score (A) and distance walked on MSWT for patient group (B), and Dyspnea-12 score plotted against distance walked on MSWT for the patient group (C). MSWT = modified shuttle walk test.

Table 1 presents participant demographics and spirometry and MSWT values. FEV_1_ did not correlate with Dyspnea-12 score or MSWT distance, but a negative correlation was observed between Dyspnea-12 score and MSWT distance (Fig 3). In patients, dyspnea and anxiety VAS scores correlated with Dyspnea-12 scores (dyspnea, *r* = 0.51, *P* = .0007; anxiety, *r* = 0.56, *P* < .0001).

### Questionnaires and VAS Ratings

**Figure 4 –:**
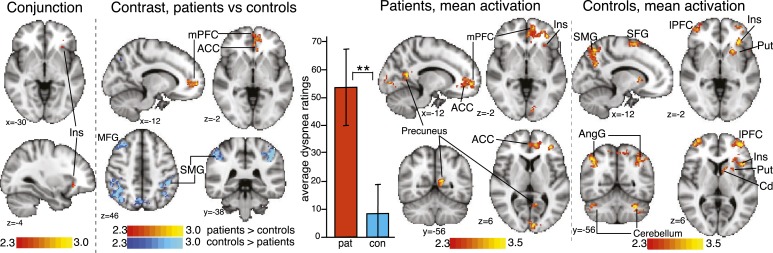
*Activation (contrasts and conjunction) correlating with VAS ratings to dyspnea word cues. Maps are cluster level corrected for multiple comparisons at *P < .05 across the whole brain. Maps represent conjunction analysis (activations common to both groups), comparisons between groups (patients > control subjects in red-yellow, control subjects > patients in blue-light blue), and mean activations in patients and control subjects. Bar graph is mean ± SD dyspnea ratings for each group. **P* < .001. Cerebellum is crus I and VI. ACC = anterior cingulate cortex; AngG = angular gyrus; Cd = caudate; con = control subjects; Ins = insula; lPFC = lateral prefrontal cortex; MFG = middle frontal gyrus; mPFC = medial prefrontal cortex; pat = patients; Put = putamen; SMG = supramarginal gyrus, SFG = superior frontal gyrus. See Figure 1 legend for expansion of other abbreviation.*

Patients scored significantly higher than control subjects on the Dyspnea-12, St. George’s Respiratory Questionnaire, Catastrophic Thinking Scale, Awareness and Vigilance Scale, Fatigue Severity Scale, Center for Epidemiologic Studies Depression Scale, and State-Trait Anxiety Inventory Scale but not on the Behavioral Inhibition System/Behavioral Activation System scale (Table 2). During FMRI, patients rated word cues higher for both dyspnea (mean ± SD: patients, 53.6 ± 13.5; control subjects, 8.4 ± 10.4; *P* < .001) and dyspnea-anxiety (patients, 43.1 ± 18.6; control subjects, 5.8 ± 10.8; *P* < .001) (Fig 4).

**TABLE 2 ]:** Questionnaire Scores

Questionnaire	Patients (n = 41)	Control Subjects (n = 40)
Dyspnea-12	13.2 ± 9.2	0.0 ± 0.0^a^
Physical	8.1 ± 5.2	0.0 ± 0.0^a^
Affective	5.1 ± 4.3	0.0 ± 0.0^a^
St. George’s Respiratory Questionnaire	52.0 ± 17.0	6.9 ± 5.1^a^
Symptom	61.8 ± 18.0	2.7 ± 2.1^a^
Activity	69.7 ± 22.4	3.1 ± 3.1^a^
Impact	39.0 ± 18.0	0.6 ± 1.4^a^
Catastrophic Thinking Scale^b^	14.5 ± 12.0	0.0 ± 0.2^a^
Helplessness	5.6 ± 5.7	0.0 ± 0.2^a^
Magnification	4.1 ± 3.1	0.0 ± 0.0^a^
Rumination	4.9 ± 4.4	0.0 ± 0.0^a^
Awareness and Vigilance Scale^c^	41.7 ± 14.6	12.9 ± 11.4^a^
Fatigue Severity Scale	42.9 ± 11.0	22.3 ± 12.0^a^
BIS/BAS	53.6 ± 8.6	54.7 ± 8.5
BAS: drive	10.0 ± 2.8	10.5 ± 2.7
BAS: fun-seeking	9.2 ± 2.5	9.4 ± 2.4
BAS: reward responsiveness	9.5 ± 2.9	9.5 ± 3.1
BIS: inhibition	16.4 ± 3.8	16.5 ± 3.7
Center for Epidemiologic Studies Depression Scale	14.8 ± 9.3	7.2 ± 6.6^a^
State anxiety	35.1 ± 9.9	25.6 ± 7.5^a^
Trait anxiety	37.6 ± 11.0	29.1 ± 6.8^a^

Data are presented as mean ± SD. Component scores are included where appropriate. BAS = Behavioral Approach System; BIS = Behavioral Inhibition System.

a *P* < .001.

b Modified from use in asthma.

c Modified from use in pain.

### Functional MRI

A full list of additional activations, their coordinates, and their *z* scores are presented in e-Tables 3, 4, and 5.

#### Correlation of FMRI Signal With Dyspnea VAS Ratings, Patients:

Activations were observed in the medial prefrontal cortex (mPFC), anterior insula, lateral prefrontal cortex (lPFC), anterior cingulate cortex (ACC), and precuneus (Fig 4).

#### Correlation of FMRI Signal With Dyspnea VAS Ratings, Control Subjects:

Activations were observed in the lPFC, anterior insula, putamen, caudate, angular gyrus, supramarginal gyrus, and superior frontal gyrus (Fig 4).

#### Correlation of FMRI Signal With VAS Ratings, Comparison of Patients and Control Subjects:

Common activation was found in the left-side anterior insula (Fig 4). A direct group comparison revealed stronger activation in the left-side mPFC and the ACC in patients; the reverse contrast showed stronger activation in the supramarginal gyrus, angular gyrus, precuneus, and middle frontal gyrus regions in control subjects.

#### Influence of Depression, Fatigue, and Vigilance on Correlation of FMRI Signal With Dyspnea VAS Ratings, Patients Only:

**Figure 5 –:**
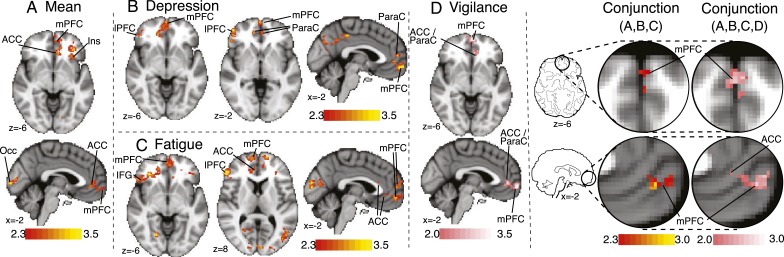
*Activations correlating with VAS ratings of dyspnea word cues for patients only. A, Mean positive activations in patients in the two slices common to B, C, and D. B-D, Activations correlating negatively with depression (B), negatively with fatigue (C), and positively with vigilance (D), respectively. Conjunction analysis represents brain areas where significant overlap exists among these activations. Maps for mean, depression, and fatigue are whole-brain analysis, cluster level corrected for multiple comparisons at* P *< .05, with a cluster threshold of *z* < 2.3, and activations are presented using standard red-yellow scaling. Activation in the vigilance contrast is presented with a cluster threshold of *z* < 2.0, and activations are presented with a pink scale to highlight this different statistical threshold. Maps represent mean correlation with respective questionnaire scores in patients only. IFG = inferior frontal gyrus; Occ = occipital cortex; ParaC = paracingulate cortex. See Figure 1 and 4 legends for expansion of other abbreviations.*

Significant negative correlations were observed between both depression and fatigue and activations in the mPFC, lPFC, and ACC. We observed clusters of subthreshold-positive activation at *z* = 2.0 for vigilance scores (Fig 5) in the mPFC and ACC. *F* tests revealed no shared variance between the contrasts. Conjunction analysis between all questionnaires and the mean activation revealed significant overlap in the mPFC and ACC (Fig 5).

#### Correlation of FMRI Signal With Anxiety VAS Ratings:

No significant mean effects were observed in either patients or control subjects. No group differences were identified.

## Discussion

In patients, we observed activation of the mPFC, lPFC, ACC, anterior insula, and precuneus that correlated with the subjective dyspnea (VAS) response to the word cues. Furthermore, some of the variability in the brain response to these word cues is explained by measures of depression, fatigue, and vigilance.

The findings in the mPFC and ACC are of particular interest because FMRI activation in these areas was stronger than in healthy control subjects (Fig 4). The mPFC has been linked with fear-related memory processes and emotional learning and is a key structure engaged in chronic but not in acute pain.^27,28^ For example, in patients with chronic back pain, acute thermal pain engages the insula, whereas spontaneous chronic pain is associated with activity in the mPFC.^27^ The mPFC is considered a key component of a chronic pain suffering model that includes the lPFC and ACC.^29^ The mPFC has not been identified in any previous FMRI studies of dyspnea; however, none examined the chronic state. Activation in the ACC has been identified in two FMRI studies of experimental dyspnea,^30,31^ but activations were not correlated with a particular psychologic measure.

Depression is a well-established major influence in dyspnea. Taking parallels from the study of pain where depression enhances pain unpleasantness through the mPFC,^32^ the present data suggest that similar mechanisms may be in play in dyspnea. Despite strong clinical associations with COPD, the brain mechanisms of fatigue on sensory processes remain poorly understood. The present data begin to elucidate this by showing that level of fatigue in patients with COPD correlates with prefrontal activation in response to dyspnea-related cues, which might indicate that fatigue influences emotional processing. Hypervigilance is similarly well described in chronic dyspnea^6,9^ and an important component of fear avoidance.^33^ Hypervigilance may amplify dyspnea perception by altering the way the mPFC and ACC respond to dyspnea-specific situations.

Activation was also observed in the lPFC, which is known for its role in cognitive decisions regarding reacting to potentially harmful stimuli.^29^ The present data suggest that fatigue and depression influence the brain’s processing of dyspnea cues by acting in this structure. This may represent the patients’ altered evaluation of the word cues in the context of real-life experiences. The lPFC has been identified in experimentally induced acute dyspnea,^30^ but this is the first time in our knowledge that it has been linked to specific behavioral measures.

Dyspnea ratings were correlated with activation in the left-side anterior insula, a structure associated with interoception, the conscious awareness of bodily sensations.^34,35^ The insula has been identified in all FMRI studies of acutely induced dyspnea.^7^ Although the overall insular activation to the word cue task was stronger in patients than in control subjects (e-Fig 2), the correlation between dyspnea VAS ratings and FMRI activations was the same (Fig 4). Taken together, these findings raise the question about whether activity in the insula is dyspnea specific or relates to more universal interoceptive processes and whether emotional engagement in dyspnea is downstream of or separate from interoception.

The findings in healthy control subjects (Fig 4) identify broadly the same brain areas as in other healthy volunteer FMRI studies of dyspnea and respiratory sensation.^7,36,37^ In the mPFC and ACC, patients with COPD demonstrate FMRI activation to these cues that is not observed in healthy control subjects. We propose that the findings relate to the different psychologic processing of these environmental cues, with greater salience and stronger negative meaning in COPD. In the everyday life of patients with COPD, these cues may be learned associations of normally innocuous scenarios of dyspnea or simply anticipation of physical activities, which may prime the brain for readiness. It is known that cues can exacerbate symptom perception in pain^38,39^ and asthma^40^ by enhancing brain activity in relevant areas before stimulus onset.^41^ The present data suggest that similar mechanisms of heightened responses to dyspnea-related environmental cues could increase the threat value or amplify the sensations of dyspnea, making dyspnea more unpleasant or more frightening.

We further speculate that engagement of these frontal areas may either lead to reorganization of the brain’s emotional circuitry (as has been proposed in chronic pain^29^) or alternatively, that these circuits are simply recruited more readily. However, further research is necessary.

The interpretation of the present imaging findings is supported by clinical evidence suggesting that emotional learning processes contribute to dyspnea. To characterize the mechanisms of emotional learning and determine the effect of chronicity, longitudinal FMRI studies are necessary. These could examine either natural change in dyspnea over time or brain changes in response to an intervention. For example, dyspnea-related fear measured before pulmonary rehabilitation correlates with improvements in dyspnea,^9^ strongly suggesting the importance of emotional learning.

### Limitations

This study used word cues to engage brain networks responsible for dyspnea processing. The approach may be more suited to interrogating the emotional-cognitive aspects that modulate dyspnea rather than those brain activations related to direct sensory input.^42,43^ It is worth noting that the word cue responses correlate with Dyspnea-12 scores, so they are likely to represent a meaningful aspect of clinical dyspnea. Healthy control subjects may interpret word cues differently, reflecting a differing response to real-life situations. Targeting such variation was the aim of the study. However, to compare absolute dyspnea between patients and control subjects, a different approach might be taken, for example, adopting the paradigm of O’Donnell et al^44^ or comparing word cues with similar dyspnea valence.

The activations relating to the vigilance contrast did not survive cluster thresholding at the standard threshold of *z* > 2.3. Although we have less confidence in this particular finding, it would be misleading to ignore it completely. More work is needed to determine the role of vigilance in COPD.

Because smoking history was strongly associated with group (ie, patients, control subjects), we did not include it as a regressor in the FMRI analysis. Smoking has known effects upon the brain, but it remains unclear whether it has specific effects on the FMRI signal. We, therefore, included a control task (random letter strings) that demonstrated no difference between the groups. We suggest that future work might compare brain responses in groups matched for smoking history.

## Conclusions

The findings suggest that emotional processes such as depression, fatigue, and vigilance play an important role in shaping the brain mechanisms associated with interpreting dyspnea-related cues in COPD. Heightened responses to salient cues are associated with increased symptom perception in other disorders, and the findings suggest that similar mechanisms may also be relevant in COPD. Engagement of these emotion-regulating areas may contribute to the poor correlation between lung function and dyspnea severity.

Future work would look in more detail at these structures and how interventions may affect dyspnea processing. Understanding the neural processing of dyspnea in a clinical population is crucial for advances to be made in its treatment, such as the development of neuroimaging biomarkers that allow patient stratification, leading to individualized treatments.

## Supplementary Material

Online Supplement

## ABBREVIATIONS

ACC: anterior cingulate cortex
FMRI: functional MRI
lPFC: lateral prefrontal cortex
mPFC: medial prefrontal cortex
MSWT: modified shuttle walk test
Sao_2_: oxygen saturation as measured by pulse oximetry
VAS: visual analog scale
