# Supplementary material for: Dyspnea-Related Cues Engage the Prefrontal Cortex: Evidence From Functional Brain Imaging in COPD
Source: Chest. 2015 Jul 2;148(4):953–61. doi: 10.1378/chest.15-0416 (PMC4594628; doi:10.1378/chest.15-0416)
Supplement: Supplementary file 1 [file chest_148_4_953_ds01.pdf]

CHEST Online Supplement

Dyspnea-Related Cues Engage the Prefrontal Cortex

Evidence From Functional Brain Imaging in COPD

Mari Herigstad, DPhil; Anja Hayen, DPhil; Eleanor Evans, MSc; Frances M. Hardinge, MD; Robert J. Davies, MD†; Katja Wiech, PhD; and Kyle T. S. Pattinson, DPhil

CHEST 2015; 148(4):953-961

e-Appendix 1.

EXTENDED METHODS SECTION

Participants

We recruited 44 patients with mild to moderate COPD in the week before commencing a course of pulmonary rehabilitation, and 40 age-and-sex matched healthy controls (see Table E5 for medical questionnaire summary). Three patients who were studied were subsequently excluded from the analysis as neither the medical history obtained, nor spirometry, indicated COPD. Two of these patients had restrictive lung disease, and in the other patient the cause of dyspnea was undefined but probably cardiac.

The analysis presented therefore includes 41 patients (15F, age 68.0+/-8.2(SD)) and 40 controls (16F, age 69.1+/-8.1(SD)). See Table 1 for GOLD staging and its relation to MRC dyspnea scores. At the time of the study, a primary care diagnosis of COPD was an entry criterion for pulmonary rehabilitation. However, based on our testing in this study, four of these patients were categorised as GOLD stage 0 (i.e. FEV1>80%), these patients were included in the analysis as they had symptomatic dyspnea, had a strong history of exposure to cigarette smoke, had been referred for pulmonary rehabilitation and had no clear alternate cause for their dyspnea.

Exclusion criteria were MRI contraindications (e.g. ferrous implants), dependency on oxygen therapy, inability to complete the research tasks, diabetes, history of stroke, opioid use and epilepsy.

†Deceased.

Online supplements are not copyedited prior to posting.

© 2015 AMERICAN COLLEGE OF CHEST PHYSICIANS. Reproduction of this article is prohibited without written permission from the American College of Chest Physicians. See online for more details. **DOI:** 10.1378/chest.15-0416

CHEST™ Online Supplement

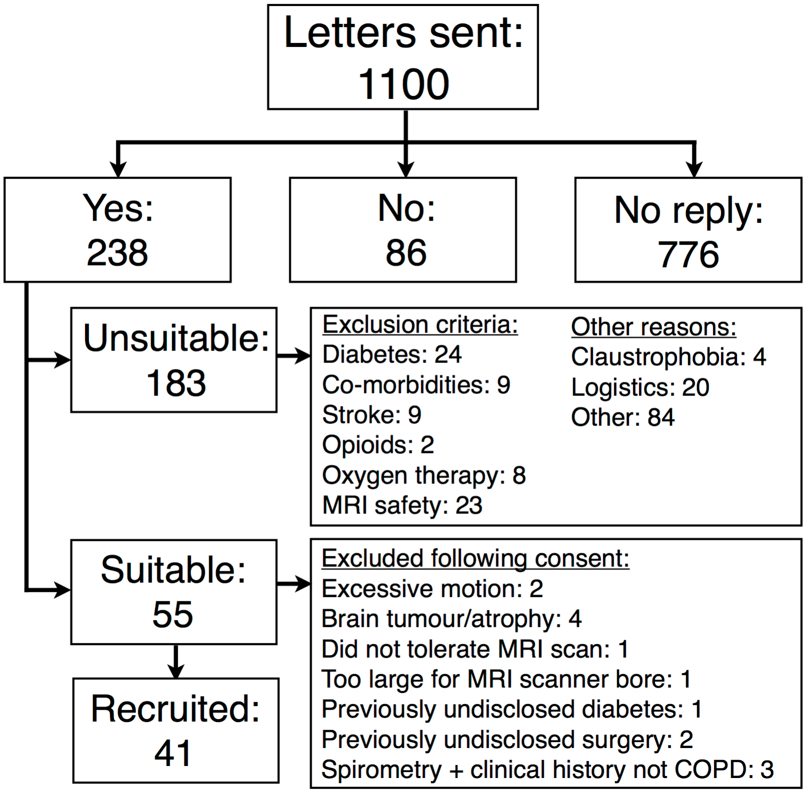

e-Figure 1: Details of recruitment numbers for COPD patients and reasons for exclusion, FMRI study. MRI safety includes all MRI contraindications, such as ferrous implants, pacemakers and large tattoos. COPD patients were recruited as part of their invitation for pulmonary rehabilitation treatment via the Oxfordshire Primary Care Trust, Oxford University Hospitals NHS Trust and Oxford Health NHS Foundation Trust. Controls were recruited through poster advertisements on public noticeboards in Oxfordshire.

Online supplements are not copyedited prior to posting.

© 2015 AMERICAN COLLEGE OF CHEST PHYSICIANS. Reproduction of this article is prohibited without written permission from the American College of Chest Physicians. See online for more details. **DOI:** 10.1378/chest.15-0416

CHEST™ Online Supplement

e-Table 1: MEDICAL QUESTIONNAIRE SUMMARY

A researcher-administrated structured interview was conducted by a trained respiratory nurse. The aim was to assess the participant's full medical, social and occupational history, as well as family history of respiratory disease. A summary of the main findings from this interview can be found below:

	**COPD patients**	**Healthy controls**
Age of COPD diagnosis (years)	59.3 (14.1) [a]	n/a
Time since last COPD exacerbation (months)	6.7(12.6) [a]	n/a
Number of exacerbations in previous year	2.1(2.1) [a]	n/a
Past medical history		
Asthma	18	3*
Bronchiectasis	3	0
Hypertension	18	6
Myocardial infarction, atrial fibrillation, angina	23	8
Osteoporosis	3	0
Irritable bowel disease, ulcers or reflux and	31	11
Depression requiring treatment	16	5
Other major psychiatric illness	0	0
Tuberculosis of the lungs	2	2
Family history of respiratory disease (parent)	16	4
Family history of respiratory disease (sibling)	4	2
Currently employed	10	12
Smoking history		
Smoker	10	0
Ex-smoker	29	19
Never smoker	2	21
Exposed to cigarette smoke at home	10	0
Exposed to cigarette smoke during childhood	37	26
Units of alcohol per week	9.2 (13.5) [a]	9.1(8.9) [a]

Values are averages (and standard deviations) †, or sums.* childhood asthma, not on any current treatment ** not formally diagnosed

Online supplements are not copyedited prior to posting.

© 2015 AMERICAN COLLEGE OF CHEST PHYSICIANS. Reproduction of this article is prohibited without written permission from the American College of Chest Physicians. See online for more details. **DOI:** 10.1378/chest.15-0416

e-Table 2: MODIFIED BORG SCALE

0	Nothing at all
0.5	Very, very slight (just noticeable)
1	Very slight
2	Slight
3	Moderate
4	Somewhat severe
5	Severe
6	
7	Very severe
8	
9	Very, very severe (almost maximal)
10	Maximal

FMRI

Imaging was performed at the University of Oxford Centre for Clinical Magnetic Resonance Research with a Siemens 3Tesla TIM Trio scanner, using a 12-channel head coil. Participants undertook two FMRI scans, each using a BOLD echo-planar image (EPI) acquisition (time repetition (TR)=3000ms, time echo (TE)=30ms, field of view 192x192mm, voxel size 3x3x3mm, 45 slices, 168 volumes). Each FMRI scan lasted 8 minutes and 20 seconds, with the break between scans allowing participants a chance to cough. A structural T1-weighted, whole-brain scan (MPRAGE sequence, TR=2040ms, TE=4.68ms, flip angle of 8°, voxel size 1.0x1.0x1.0mm) was obtained and used for image registration. Scans were always performed in the same order.

During the FMRI scans, participants were presented with a set of randomised dyspnea-related cues (24 in total) on a screen (white text, black background; displayed for 7 seconds). Dyspnea-related cues ranged from near-neutral (e.g. 'Sitting in a chair') to strenuous/distressing scenarios (e.g. 'Walking uphill') and were developed in a pilot trial in a separate group of COPD patients. Participants were asked to rate each cue, first according to how breathless they would feel ('How breathless would this make you feel?') and second how anxious they would feel ('How anxious would this make you feel?'), in each given scenario on a visual analogue scale (VAS) scale (range:0-100; anchors: 'Not at all' and 'Very much', 7 seconds). Ratings were always in the same order. A control task to assess potential differences in baseline BOLD responsiveness between groups was employed in the form of random letter strings which were presented after every third dyspnea-related word cue. These were not followed by any ratings and participants were instructed to ignore them. Participants were trained to do the task immediately prior to the scan, using a

Online supplements are not copyedited prior to posting.

© 2015 AMERICAN COLLEGE OF CHEST PHYSICIANS. Reproduction of this article is prohibited without written permission from the American College of Chest Physicians. See online for more details. **DOI:** 10.1378/chest.15-0416

different set of cues. Training was repeated until participants could complete the task reliably on their own. Participants were instructed to keep their eyes open for the full duration of the BOLD sequences.

Data analysis

All FMRI data processing was carried out within FEAT (FMRI Expert Analysis Tool) Version 5.98, part of FSL (Oxford Centre for Functional Magnetic Resonance Imaging of the Brain (FMRIB) Software Library, version 5.0 www.fmrib.ox.ac.uk/fsl).

Prestatistic processing of the data included MCFLIRT motion correction,[1] removal of nonbrain structures,[2] spatial smoothing (full-width-half-maximum Gaussian kernel of 5mm) and high-pass temporal filtering (Gaussian-weighted least-squares straight line fitting, high-pass filter cut-off of 90s). First-level data were modeled (voxel-by-voxel) using FMRIB's Improved Linear Model (FILM) with local autocorrelation correction.[3] FSL Motion Outliers was used to detect timepoints in the dataset subject to large, rapid motion artefacts (such as coughs). A confound matrix was generated and used in the general linear model (GLM) to remove the effects of these timepoints. Physiological measurements of the respiratory cycle and pulse oximetry during the scan were used to account for respiratory- and cardiovascular-related noise effects, using RETROICOR,[4,5] with 4 cardiac, 4 respiratory, 2 interaction terms and respiratory-volume-time (RVT) smoothing of 15.

Images were then registered to the MNI152 standard space using an affine registration (FMRIB Linear Image Registration Tool, FLIRT) between the EPI and T1 structural scan and a nonlinear registration (FNIRT) between T1 structural scans and the MNI standard brain.

First-level analyses used a general linear model (GLM) with multiple explanatory variables (EVs) which were: presentation of word cues, trial-by-trial dyspnea and anxiety ratings of word cues, random letter strings and periods when subjects were rating using the VAS in both patients and controls. The GLM assumed a 6-second haemodynamic delay. Contrast images were calculated as appropriate and used for higher-level analyses.[6]

In ten cases, individuals rated their anxiety consistently at zero, setting contrasts involving the anxiety EV to zero in the first-level analysis. Higher-level FEAT analysis does not automatically omit contrasts that involve empty EVs but rather inserts a parameter estimate image with the value zero, which is not an accurate representation of an empty EV. Therefore, contrasts for such empty EVs were manually removed from higher-level analyses of anxiety ratings.

Online supplements are not copyedited prior to posting.

© 2015 AMERICAN COLLEGE OF CHEST PHYSICIANS. Reproduction of this article is prohibited without written permission from the American College of Chest Physicians. See online for more details. **DOI:** 10.1378/chest.15-0416

CHEST Online Supplement

For all lower-level analyses, the two scans for each participant were combined using a fixed-effects model. This was done by forcing the random effects variance to zero in FLAME (FMRIB's Local Analysis of Mixed Effects).[6-8] Inputs were lower-level FEATs (two per participant). Each contrast of parameter estimates (COPE) image from this second-level analysis could then be entered into a third, higher-level analysis to examine group differences.

A higher (group) level mixed-effects analysis was performed, to compare brain activation across groups using FLAME 1+2.[7] The COPEs analysed were the presentation of dyspnea-related cues and random letter strings from the first lower-level analysis, and breathlessness and anxiety ratings of the dyspnea-related cues from the second lower-level analysis.

As fatigue, depression and vigilance are known to be major factors in dyspnea these were considered regressors of interest. As state anxiety may have been confounded by experimental factors this was considered a regressor of no interest. We had no prior expectation of any link between these factors and breathlessness in controls, and these regressors were therefore not further interrogated in the control group.

To correct for multiple comparisons, Z statistic images were thresholded using clusters determined by $Z > 2.3$ and a (corrected) cluster significance threshold of $p=0.05$ across the whole brain.[9] To detect important sub-threshold activations, the analysis was then repeated in which Z statistic images were thresholded using clusters determined by $Z > 2.0$ and a (corrected) cluster significance threshold of $p=0.05$ across the whole brain.

We performed conjunction analyses (conjunction null) to determine common areas of mean brain activation in each group and between the questionnaire regressors in patients only. F-tests examined shared variance between these questionnaire-based regressors.

To account for potentially confounding differences in psychological functioning, behavioural measures were also added to the GLM as additional regressors: State Anxiety, Fatigue Severity Scale, CES-D and Awareness and Vigilance Scale. These were identified using multiple regression analysis (IBM SPSS Statistics) with dyspnea scores obtained through the Dyspnoea-12 questionnaire as the dependent variable and other questionnaire scores as independent variables (see next section of online supplement).

Online supplements are not copyedited prior to posting.

© 2015 AMERICAN COLLEGE OF CHEST PHYSICIANS. Reproduction of this article is prohibited without written permission from the American College of Chest Physicians. See online for more details. **DOI:** 10.1378/chest.15-0416

The Dyspnoea-12 was chosen as it is a validated, dyspnea-specific measure of respiratory symptoms in COPD. Variables with a variable inflation factor above five were excluded from the regression analysis.

We did not include pack-years as a nuisance regressor because this variable is strongly linked to group, i.e. the COPD patients had substantial smoking history and the healthy controls did not. As smoking (and COPD) has unknown effects on BOLD responsiveness, we included a control task (random letter strings) to specifically test whether the BOLD response behaved similarly in both groups (described above).

Two main group level analyses were conducted.

- Main analysis: EVs for patients and controls were modelled separately, accounting for any differences in the linearity of response between patients and controls. As fatigue, depression and vigilance are known to be major factors in dyspnea these were considered regressors of interest. As state anxiety may have been confounded by experimental factors this was considered a regressor of no interest. We had no prior expectation of any link between these factors and breathlessness in controls, these regressors were not further interrogated.
- A supplementary basic analysis (presented only in the supplementary material) was also performed in which the EVs included the presentation of dyspnea-related word cues (across the whole range from low to high valence), random letter strings and the periods when subjects were rating dyspnea (VAS scale presentation) but without VAS scores or questionnaire scores.

Conjunction analyses (conjunction null) were performed to determine common areas of brain activation across groups, and unpaired t-tests were used to determine group differences.

To assess group differences in generalised BOLD responsiveness, a region-of-interest (ROI) analysis explored between-group differences in the BOLD response to visual stimulation for random letter strings. A ROI (visual cortex, bilateral V1) mask was created using a standard atlas (Juelich Histological Atlas, thresholded at 50%) and used to identify the average signal change within this mask for all participants [10] in response to the random letter strings (visual stimulus). Student's T-test was then used to assess group differences in visual stimulus-induced BOLD response.

Identification of additional regressors for group analysis

Patients show significantly higher psychomorbidity than the general population and such psychological factors are interlinked with the dyspnea symptom. In order to address effects of psychological factors, we

© 2015 AMERICAN COLLEGE OF CHEST PHYSICIANS. Reproduction of this article is prohibited without written permission from the American College of Chest Physicians. See online for more details. **DOI:** 10.1378/chest.15-0416

included selected questionnaire scores as additional regressors in the higher level analysis. To choose the most relevant behavioural measures, a multiple regression analysis was used to identify psychological factors predicting dyspnea levels (measured by the Dyspnoea-12 questionnaire, as it is a measure specific for both dyspnea and COPD). These scores were then added as additional regressors in the group analysis.

The Dyspnoea-12 questionnaire contains a total score and two sub-scores measuring physical aspects and affective aspects of dyspnea. Each score was used as a dependent variable in a separate multiple linear regression (IBM SPSS Statistics). Candidate models were fitted separately and then combined. Independent variables were behavioural measures.

Potential inter-correlations were controlled for as follows: To ensure that our set of predictors was not weakened by unacceptably high levels of intercorrelation, we first employed a cut-off level of $r>0.8$ for correlation between measures. That led to the exclusion of the trait anxiety questionnaire. We then performed a multiple regression analysis in SPSS and checked co-linearity diagnostics. Variance inflation factors (VIFs), tolerance values and condition indices were checked. VIFs over 5 were considered unacceptable, as were condition indices over 15 (paired with high variance proportions (0.5 or larger)) and tolerance of less than 0.2.

Interrogating VIFs and tolerance values examines the independent variable in relation to all other independent variables, and interaction effects are therefore taken into account. This provides a more robust exclusion parameter than simple correlations. Independents that conformed to these cut offs were dropped. The remaining independents were included in the final multiple regression analysis. None of the independents that made this final set of predictors employed VIFs, condition indices or tolerance values above the above-cited cut offs.

The prediction model for the total score of the Dyspnoea-12 questionnaire accounted for 66.0% of the variance of the score ($R2=0.674$, adjusted $R2=0.660$ and was statistically significant ($F(3,74)=50.9, p<0.01$). The model contained 3 of 6 predictors, with the primary predictor being higher values on the Awareness and Vigilance Scale (beta: 0.369) followed by higher values on the CES-D (beta: 0.386) and Fatigue Severity Scale (beta: 0.237). The prediction models for the component scores showed the following: physical component $R2=0.674$ (adjusted $R2=0.656$), $p<0.001$ ($F(4,73)=37.8$), containing 4 of 6 predictors (higher values on the Awareness and Vigilance Scale (beta: 0.442), State Anxiety Inventory (beta: 0.171, CES-D (beta: 0.212) and Fatigue Severity Scale (beta: 0.188)); affective

Online supplements are not copyedited prior to posting.

© 2015 AMERICAN COLLEGE OF CHEST PHYSICIANS. Reproduction of this article is prohibited without written permission from the American College of Chest Physicians. See online for more details. **DOI:** 10.1378/chest.15-0416

component $R2=0.639$ (adjusted $R2=0.624$), $p<0.001$ ($F(3,74)=43.6$) containing 3 of 6 predictors (higher values on the CES-D (beta: 0.456), Awareness and Vigilance Scale (beta: 0.268) and Fatigue Severity Scale (beta: 0.240)).

Analysis of respiratory pattern in the patient group.

A separate assessment of the respiratory data was also conducted to investigate whether COPD patients altered their breathing in response to dyspnea-related cues. Respiratory bellows systems measure the tension produced by the change in chest circumference with each breath. While chest expansion cannot account for lung expansion into the abdomen, it nevertheless provides a representation of tidal volume. Respiratory rate and chest expansion were thus calculated from the individual respiratory bellow traces and interpolated over the scan sessions (one data point per second) using custom-made Matlab scripts (Mathworks Inc., US). Respiratory rate multiplied by chest expansion did not change significantly neither between the presentation of dyspnea-related word cues and random letter strings ($p=0.98$), nor the presentation of dyspnea-related word cues and fixation crosses ($p=0.96$) in COPD patients.

Comparison of generalised BOLD responsiveness between patients and controls

Visual stimuli may be used to address differences in BOLD responsiveness between groups. BOLD FMRI relies on a relative change in paramagnetic deoxy-hemoglobin concentration. Certain conditions (e.g. hypertension, stroke) may potentially alter the coupling of cerebral blood flow and blood volume to metabolic rate, thus changing/abolishing the BOLD signal. This baseline difference in BOLD responsiveness in the patient group might be taken as a 'real' group difference in neuronal activation. At the time of writing there were only three other published neuroimaging studies in COPD patients (none of which address respiratory sensations), and little was known about BOLD responsiveness in this patient population. As such, it was possible that neurovascular coupling could be different in COPD patients due to disease process or drugs, even when correcting for age and sex.

We would expect baseline differences in BOLD responsiveness to manifest as group differences across the whole brain rather than a subset of regions linked by function (anxiety responses). Therefore, to investigate whether changes in BOLD signal observed arose from 'real' differences in neuronal activation, we interrogated the response to random letter strings specifically in the visual cortex in both groups.

The average activation level of the voxels within the visual cortex (V1) did not differ significantly between groups (mean signal change 0.52% (SD 0.28%) in patients, 0.54% (SD 0.26%) controls $p=0.69$). This suggests that there was no significant between-group difference in BOLD responsiveness, or that BOLD

© 2015 AMERICAN COLLEGE OF CHEST PHYSICIANS. Reproduction of this article is prohibited without written permission from the American College of Chest Physicians. See online for more details. **DOI:** 10.1378/chest.15-0416

responsiveness in COPD patients is altered to a lesser degree than is detectable by our significance criteria, and that BOLD signal changes in the present study thus reflect actual differences in neuronal activation.

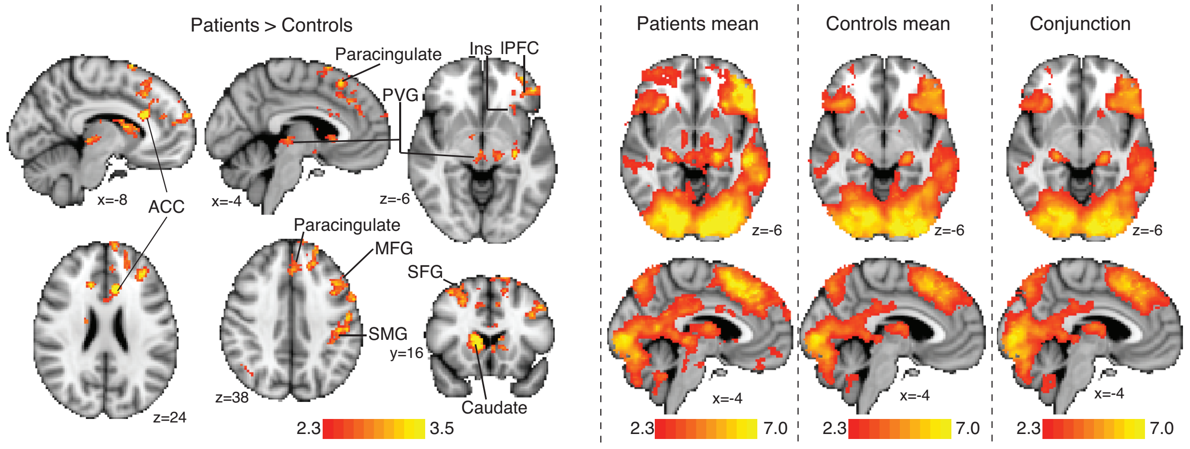

e-Figure 2: Activation during the presentation of words for patients and controls, not scaled by VAS ratings. Maps are whole-brain analysis, cluster level corrected for multiple comparisons at $p<0.05$. Maps represent comparison between groups (patients>controls), patient mean, controls mean, and conjunction as labelled. No activation was greater in controls than in patients. Abbreviations: ACC (anterior cingulate cortex), PVG (periventricular grey), Ins (insula), lPFC (lateral prefrontal cortex), MFG (middle frontal gyrus), SFG (superior frontal gyrus), SMG (supramarginal gyrus).

Supplementary analysis: results

Both groups exhibited bilateral activation in response to word cues in an extended network comprising illustrated above (Fig E2) and in Table E4. Compared to healthy controls, the patient group showed stronger activation in the left lateral PFC, supramarginal gyrus and insular cortex, and bilateral anterior cingulate cortex (ACC), caudate, thalamus and periventricular/periaqueductal grey (PVG/PAG). In no brain regions did healthy controls show stronger activation than patients.

Supplementary analysis: discussion

Patients demonstrated greater activation in the left dorsal and ventrolateral PFC, insular cortex, ACC, PVG/PAG and thalamus, which are areas known to be involved in anxiety, threat and fear processing. Abnormal insular activity is observed in patients with anxiety disorders,[11] and insular activation is

Online supplements are not copyedited prior to posting.

© 2015 AMERICAN COLLEGE OF CHEST PHYSICIANS. Reproduction of this article is prohibited without written permission from the American College of Chest Physicians. See online for more details. **DOI:** 10.1378/chest.15-0416

associated with negative emotions in experimentally-induced dyspnea.[12] The ACC[11,13] and PFC[13] are implicated in the appraisal of threat associated with pain and noxious respiratory challenges. The PVG and PAG are associated with anxiety and fight-or-flight escape behaviours.[14] The pattern also broadly reflects brain areas activated in FMRI studies of experimentally-induced dyspnea.[12] These findings are plausible in as much as patients demonstrated higher levels of generalised anxiety (STAI questionnaires) and higher levels of specific dyspnea-related anxiety (VAS recordings).

Online supplements are not copyedited prior to posting.

© 2015 AMERICAN COLLEGE OF CHEST PHYSICIANS. Reproduction of this article is prohibited without written permission from the American College of Chest Physicians. See online for more details. **DOI:** 10.1378/chest.15-0416

e-Table 3: FMRI activation, group interactions, Correlation of FMRI signal with VAS rating of dyspnea word cues.

Contrast	Region	Peak voxel location X,Y,Z (z max) Left	Right
**Patients > Controls**			
	medial prefrontal cortex	-12, 58, 0 (3.13)	
	medial prefrontal cortex	-16, 48, 2 (3.08)	
	anterior cingulate cortex	-6, 32, -6 (3.08)	
	lateral prefrontal cortex	-36, 42, -2 (3.77)	
	precuneous	-12, 58, 14 (3.72)	
**Controls > Patients**			
	medial frontal gyrus		36, 32, 44 (3.95)
	lateral occipital cortex		14, -70, 58 (3.42)
	angular gyrus		40, -52, 46 (3.81)
	supramarginal gyrus		54, -42, 52 (3.67)
	superior parietal lobule	-44, -40, 48 (4.14)	
	cerebellum, crus 1	-34, -60 , -40 (3.8)	
**Patients mean**			
	lateral prefrontal cortex	-36, 40, -4 (4.22)	
	medial prefrontal cortex	-12, 56, -4 (3.52)	
	paracingulate	-10, 46, 6 (3.48)	
	anterior cingulate	-6, -34, 5 (2.79)	
	insula	-30, 26, 0 (2.46)	
	precuneous	-12, -58, 14 (3.72)	
	occipital pole	-4, -94, 4 (4.13)	
**Controls mean**			
	lateral prefrontal cortex	-38, 54, 4 (4.37)	36, 58, 2 (4.31)
	paracingulate gyrus	-6, 30, 40 (3.6)	
	frontal orbital cortex	-34, 32, 2 (4.61)	
	insula	-32, 18, -8 (2.85)	
	frontal operculum	-40, 18, 6 (3.61)	
	caudate	-14, 14, 4 (3.79)	
	putamen	-26, 10, 4 (3.18)	
	superior parietal lobule	-38, -50, 58 (4.89)	
	supramarginal gyrus		48, -40, 52 (4.33)
	superior frontal gyrus	-22, 10, 54 (4.43)	
	cerebellum crus 1	-32, -56, -34 (4.11)	34, -66, -34 (3.73)

Peak voxel locations and peak z scores (zmax) during FMRI, MNI standard space.

Online supplements are not copyedited prior to posting.

© 2015 AMERICAN COLLEGE OF CHEST PHYSICIANS. Reproduction of this article is prohibited without written permission from the American College of Chest Physicians. See online for more details. **DOI:** 10.1378/chest.15-0416

CHEST™ Online Supplement

e-Table 4: FMRI activation, Correlation of FMRI signal with VAS rating of dyspnea word-cues, questionnaire contrasts, patients only.

Contrast	Region	Peak voxel location X,Y,Z (z max) Left	Right
**Depression**			
	medial prefrontal cortex	0, 58, -10 (-4.75)	
	anterior cingulate cortex		2, 36, -8 (-2.77)
	paracingulate cortex	0, 52, 10 (-3.39)	
	lateral prefrontal cortex		46, 52, 0 (-4.09)
	supramarginal gyrus		42, -36, 38 (-4.09)
	precuneous cortex	-8, -48, 38 (-3.54)	
	inferior frontal gyrus		50, 10, 18 (-3.84)
	supramarginal gyrus	-38, -48, 42 (-3.73)	
	lateral occipital cortex		44, -72, 30 (-3.46)
**Fatigue**			
	medial prefrontal cortex		8, 60, 12 (-4.43)
	anterior cingulate cortex	0, 26, 20 (-2.85)	6, 42, 14 (-3.11)
	lateral prefrontal cortex		46, 40, 22 (-3.73)
	inferior frontal gyrus		50, 30, -2 (-4.27)
	lateral occipital cortex	-42, -76, 0 (-3,58)	
	occiptial pole		18, -90, 16 (-4.3)
**Conjunction: mean, depression, fatigue**			
	medial prefrontal cortex	-2, 54, -8 (-2.87)	
	anterior cingulate cortex / paracingulate cortex	-2, 50, -4 (-2.41)	
Vigilance (cluster threshold z>2.0)			
	medial prefrontal cortex	*-4, 62, -4 (3.89)*	
	anterior cingulate cortex / paracingulate cortex	*-4, 46, 0 (3.29)*	
Conjunction: mean, depression, fatigue, vigilance (cluster threshold z>2.0)			
	medial prefrontal cortex	*-2, 62, -4 (2.5)*	
	anterior cingulate cortex	*0, 44, 2 (2.2)*	

Peak voxel locations and peak z scores (z max) during FMRI, MNI standard space.

Online supplements are not copyedited prior to posting.

© 2015 AMERICAN COLLEGE OF CHEST PHYSICIANS. Reproduction of this article is prohibited without written permission from the American College of Chest Physicians. See online for more details. **DOI:** 10.1378/chest.15-0416

e-Table 5: Results of basic analysis. FMRI activation to word cues without weighting by VAS ratings or questionnaires.

Contrast	Region	Peak voxel location X,Y,Z (z max) Left	Right
**Patients > Controls**			
	lateral prefrontal cortex	-38, 48, -12 (4.96)	
	medial prefrontal cortex	-22, 46, 32 (3.39)	
	anterior cingulate	-8, 24, 24 (4.27)	
	caudate	-8, 8, 12 (3.62)	12, 16, 10 (4.54)
	insula / operculum	-32, 24, 6 (3.23)	
	periventricular gray		6, -26, -6 (2.89)
	thalamus	-14, -22, -2 (3.56)	8, -22, -2 (2.89)
	Angular gyrus		
	Middle frontal gyrus		30, 22, 54 (3.69)
	Superior parietal lobe	-34, -44, 62 (4.96)	
**Patients and controls conjunction**			
	Visual cortex		12, -88, 2 (8.07)
	frontal orbital cortex	-32, 26, 0 (7.89)	34, 26, 2 (6.41)
	cerebellar vermis		2, -54, -36 (4.9)
	middle temporal gyrus		58, -38, -10 (3.35)
	lateral geniculate body		24, 24, -4
	cerebellum crus x		22, -40, -44
	temporal fusiform cortex	-40, -14, -22 (2.78)	
	superior parietal lobe	-30, -58, 48 (6.3)	
	superior frontal gyrus	-6, 20, 52 (6.9)	
	caudate	-12, 8, 12 (5.66)	
	lateral occipital cortex		34, -66, 30 (4.62)

Peak voxel locations and peak z scores (z max) during FMRI, MNI standard space.

Online supplements are not copyedited prior to posting.

© 2015 AMERICAN COLLEGE OF CHEST PHYSICIANS. Reproduction of this article is prohibited without written permission from the American College of Chest Physicians. See online for more details. **DOI:** 10.1378/chest.15-0416

REFERENCES

1 Jenkinson M, Bannister P, Brady M, et al. Improved optimization for the robust and accurate linear registration and motion correction of brain images. Neuroimage 2002; 17:825-841

2 Smith SM. Fast robust automated brain extraction. Hum Brain Mapp 2002; 17:143-155

3 Woolrich MW, Ripley BD, Brady M, et al. Temporal autocorrelation in univariate linear modeling of FMRI data. Neuroimage 2001; 14:1370-1386

4 Harvey AK, Pattinson KT, Brooks JC, et al. Brainstem functional magnetic resonance imaging: disentangling signal from physiological noise. J Magn Reson Imaging 2008; 28:1337-1344

5 Brooks JC, Faull OK, Pattinson KT, et al. Physiological noise in brainstem FMRI. Front Hum Neurosci 2013; 7:623

6 Beckmann CF, Jenkinson M, Smith SM. General multilevel linear modeling for group analysis in FMRI. Neuroimage 2003; 20:1052-1063

7 Woolrich MW, Behrens TE, Beckmann CF, et al. Multilevel linear modelling for FMRI group analysis using Bayesian inference. Neuroimage 2004; 21:1732-1747

8 Woolrich M. Robust group analysis using outlier inference. Neuroimage 2008; 41:286-301

9 Worsley KJ, Evans AC, Marrett S, et al. A three-dimensional statistical analysis for CBF activation studies in human brain. J Cereb Blood Flow Metab 1992; 12:900-918

10 Mitsis GD, Iannetti GD, Smart TS, et al. Regions of interest analysis in pharmacological fMRI: how do the definition criteria influence the inferred result? Neuroimage 2008; 40:121-132

11 Paulus MP, Stein MB. An insular view of anxiety. Biol Psychiatry 2006; 60:383-387

12 Herigstad M, Hayen A, Wiech K, et al. Dyspnoea and the brain. Respir Med 2011; 105:809-817

13 Kalisch R, Wiech K, Critchley HD, et al. Levels of appraisal: a medial prefrontal role in high-level appraisal of emotional material. Neuroimage 2006; 30:1458-1466

14 Brandao ML, Troncoso AC, de Souza Silva MA, et al. The relevance of neuronal substrates of defense in the midbrain tectum to anxiety and stress: empirical and conceptual considerations. Eur J Pharmacol 2003; 463:225-233

Online supplements are not copyedited prior to posting.

© 2015 AMERICAN COLLEGE OF CHEST PHYSICIANS. Reproduction of this article is prohibited without written permission from the American College of Chest Physicians. See online for more details. **DOI:** 10.1378/chest.15-0416
